# Mental health during societal crisis: anxiety, depression, and stress among students at the Arab American University

**DOI:** 10.3389/fpsyg.2026.1895781

**Published:** 2026-08-03

**Authors:** Mersal Abdullah Sulaiman Mersal, Madleen Masaeed

**Affiliations:** 1 Department of Sports, Faculty of Physical Education, An-Najah National University, Nablus, Palestine; 2 Department of Education Sciences, Faculty of Arts, Arab American University, Jenin, Palestine

**Keywords:** academic performance, anxiety, depression, mental health, stress

## Abstract

**Inroduction:**

This study examined the levels of depression, anxiety, and stress among undergraduate students at the Arab American University in Palestine.

**Methods:**

A cross-sectional quantitative design was employed using the Depression Anxiety and Stress Scale (DASS-42). The sample comprised 384 undergraduate students from different academic disciplines. Data were analysed using descriptive statistics, Mann–Whitney U tests, Kruskal–Wallis H tests, and post-hoc pairwise comparisons where appropriate.

**Results:**

A substantial proportion of students were classified within the extremely severe depression category (67.45%), severe anxiety category (33.33%), and severe stress category (47.92%). A statistically significant gender difference was found in depression scores, U = 15,515.00, *p* = 0.009, with male students showing higher mean ranks than female students. Depression scores also differed significantly across GPA categories, H(3) = 8.21, *p* = 0.042. *Post-hoc* comparisons showed a significant difference between students with GPAs of 2.33–2.99 and 3.67–4.00. No statistically significant differences were found for anxiety or stress according to gender, faculty type, or GPA category.

**Discussion:**

The findings indicate a substantial burden of psychological distress within the study sample. Although causal factors were not investigated, the results support further research examining the influence of ongoing socio-political conditions and highlight the importance of appropriate university-based mental health support.

## 1 Introduction

University life serves as a significant shift in a student’s personal development and academic context. This stage of development is distinctly different from school life because it requires independent thinking, advanced methods of studying, and many students will be engaging socially more than they have during their lives. Each student articulates their own hopes and dreams and a student will need to think what hopes and dreams are going to coalesce with the skills obtained to achieve academic and personal success. There are many different experiences that students will have during this time in their lives (both positive and negative) all directed at testing their ability to adapt, engage with others, and grow resilient. The ability to feel happy and satisfied and to make sound decisions will be essential parts of the university experience. All of these feelings are highly correlated to academic success and general mental well-being (Badawi and Dabbar, 2023). In this context, and as an important aspect of university life, is the idea of mental health as a part of students’ lives. Mental health is described as a dynamic state of being, which reflects the person’s ability to cope with stress and to maintain psychological balance (Galderisi et al., 2015). Sound mental health improves productivity, learning, and social development. On the other hand, poor mental health reduces performance and increases anxiety, depression, and stress (Royal College of Psychiatrists, 2021). Accordingly, mental health has been recognised as part of global health and wellbeing priorities, including the United Nations Sustainable Development Goals (2015), particularly Goal 3: “Good Health and Well-being.”

Recent research indicates that psychological distress is highly prevalent among Palestinian university students. Ahmed et al. (2024) found that the prevalence of depressive symptoms among students attending Al-Quds, Hebron, and Najah universities was 65.9% while 60.9% of students reported anxiety. Al-Dabbour et al. (2025) also reported high levels of depression, stress, sleep impairment, and dissatisfaction with life among medical students in Gaza. Amro (2024) found PTSD and anxiety in nursing students at Palestine Polytechnic University, while Ghanim et al. (2022) reported moderate to severe psychological symptoms after COVID-19 that interfered with students’ academic participation and daily functioning. In the recent 2023 Gaza war, Amro et al. (2025) found PTSD among Al-Quds University students that related to violent invasion, displacement, and social disintegration. El-Khodary and Aboudagga (2025) also reported that academic stressors (e.g., unstable internet, electricity supply, and limited online access) significantly predicted depression and anxiety symptoms among medical students in Gaza.

Recent studies also highlight the cumulative effect of crisis conditions on Palestinian students’ wellbeing. According to Sabbah et al. (2025), approximately 40% of West Bank students experienced average “psychache” or moderate psychological pain. Qutishat (2025) stated that instability, conflict, and disruption all contribute to students’ threatening psychological sustainability, or wellbeing. Aqtam (2025) reported rates of depression at 45%, and anxiety at 37%, with participants often reporting symptoms resembling PTSD. According to Hamdan et al. (2025), 23.1% of medical students experienced moderate to severe depression and 46.8% exhibited increased anxiety. Mental health, then, is an important indicator of a person’s ability to balance responsibilities, foster relationships, and seek to reach goals. In university contexts, mental health is closely linked to students’ attention, persistence, academic engagement, and overall educational success; poor mental health may negatively affect the student experience and has also been associated with attrition and dropout (Osborn et al., 2022; Pascoe et al., 2020; World Health Organization, 2022). Taken together, these recent studies demonstrate that student mental health in Palestine remains a current and urgent research priority, particularly during periods of political violence, educational disruption, and prolonged social uncertainty.

Conceptually, mental health has been defined from a variety of perspectives. Bouhafs and Azarn (2023) noted, based on Adolf Meyer’s theory of mental health, it is the capacity to adapt to external conditions and stagnate the development of disorders by exhibiting healthy social and personal behaviours. Al-Qas and Ben Ghadhafa (2022) defined it as an ability to cope effectively with everyday challenges, while Maheria (2021) associated mental health to being in psychological harmony and inner peace. Positive mental health arises from many characteristics, including hope, goal setting, emotional regulation, and social support (Laranjeira and Querido, 2022; Carvalho et al., 2022; Sienra-Monge et al., 2023; Dong et al., 2023).

Anxiety, depression, and academic stress are among the most prevalent mental health concerns in higher education. Anxiety, a normal reflex to perceived danger, can develop into chronic disorder when anxiety becomes persistent, resulting in fear, apprehension, insomnia, and muscle tension (Fouaghla and Dhafari, 2024; Barlow, 2024; Preston et al., 2021; Al-Bahnasawy et al., 2023; Al-Zahrani and Al-Enezi, 2022; Baghdad and Al-Ashmawy, 2020; Al-Sahtoohi, 2020). Chronic anxiety may stem from psychological factors, including negative thought patterns (Al-Jabali, 2016), and health-related issues (Al-Sadoun, 2017). Depression is considered to frequently follow anxiety and is characterized by sadness, social withdrawal, and loss of interest (Clack and Ward, 2019; World Health Organization, 2021). In its mood disorder form, it may be accompanied by emotional, cognitive, and physiological symptoms, which may include fatigue, hopelessness, guilt, and suicidal ideation (Ismail, 2018; World Health Organization, 2023; Cho et al., 2019; Ismail and Ismail, 2019; Ormel et al., 2019; Abu Al-Nour et al., 2020; Al-Sayed and Sabri, 2022). Academic stress, on the other hand, is when a student receives internal and external pressure related to their college course work, exams, and performance (Al-Qarala and Al-Horai, 2024; Mukhtar, 2023; American Psychological Association, 2020). It presents itself physically, psychologically, and behaviourally and is often compounded by societal, familial, and financial pressures (Beharu, 2018; White et al., 2021; Noreen et al., 2021; Harb, 2018).

These findings underscore the necessity of examining mental health in the context of broader societal crises in Palestine, specifically among students at Arab American University. Understanding the levels of anxiety, depression, and stress among Arab American University students can further enhance our understanding of the educational and psychological difficulties, and may help inform appropriate campus-based interventions and systems of support. As such, the aim of this study is to describe the dimensions of mental health amongst Arab American University students for the purpose of producing some evidence to both contribute to the literature and inform stakeholders in Palestinian higher education in developing policy and programmes that support resilience and well-being for students.

This study contributes to the literature by focusing on undergraduate students in the Palestinian higher education context, where academic demands intersect with prolonged socio-political instability, economic uncertainty, and exposure to collective crisis. By assessing depression, anxiety, and stress among students at the Arab American University using the DASS-42, the present study provides institution-specific evidence that may inform campus-based psychological support, academic counselling, preventive interventions, and student mental health policy.

Despite the growing body of research on Palestinian students’ mental health, institution-specific evidence remains needed to understand the levels of depression, anxiety, and stress among students at the Arab American University and to inform campus-based support services.

This study seeks to address the following questions:

- What are the levels of depression, anxiety, and stress among undergraduate students at the Arab American University based on the DASS-42?
- Do depression, anxiety, and stress scores differ significantly according to gender, faculty type, and GPA category?

## 2 Methods

### 2.1 Study design and procedure

This study employed a quantitative, cross-sectional design to examine the prevalence of depression, anxiety, and stress among undergraduate students and their associations with demographic factors and academic performance. Data were collected between October and November 2025. Eligible participants were undergraduate students enrolled at the Arab American University, aged 18 years or older, and willing to participate voluntarily. Students who submitted incomplete questionnaires or inconsistent responses were excluded from the final analysis.

The questionnaire was administered to students using a structured survey format. Before completing the questionnaire, participants were informed about the purpose of the study, the voluntary nature of participation, the confidentiality of their responses, and their right to withdraw at any stage. The survey consisted of two sections: demographic and academic variables, including gender, faculty type, and GPA category; and the DASS-42 items measuring depression, anxiety, and stress. A total of 384 valid responses were retained for analysis after excluding incomplete or inconsistent entries.

### 2.2 Study population and sample

The population for this study included all undergraduate students attending the Arab American University, which had a total of 11,618 students according to official university data (Arab American University, n.d.). The minimum required sample size was estimated using Cochran’s finite population sample-size formula (Cochran, 1977), based on a total undergraduate student population of 11,618, a 95% confidence level, a 5% margin of error, and a 50% response distribution. The estimated minimum required sample size was 372 students. The final sample included 384 valid responses, which exceeded the minimum requirement. Therefore, the sample size was considered adequate for the descriptive analyses and non-parametric group-comparison tests conducted in this study.

A convenience sampling approach was used because it allowed access to students from different faculties within the university during the data collection period. Although this approach enabled the researcher to obtain the required sample size, it may limit the generalisability of the findings beyond the study setting.

### 2.3 Data collection instrument

#### 2.3.1 Mental health scale

The assessment consisted of two sections. The first section gathered demographic information including grade point average (GPA), gender, and faculty type to make comparisons across different groups.

The second section assessed depression, anxiety, and stress using the Depression Anxiety Stress Scales-42 (DASS-42), originally developed by Lovibond and Lovibond (1995). The Arabic version of the DASS-42 has demonstrated acceptable psychometric properties in previous validation research (Moussa et al., 2017). The scale consists of 42 items distributed equally across three subscales, with 14 items for each subscale. The original DASS-42 response format was used without modification. Each item was scored on a four-point scale ranging from 0 to 3, where 0 indicates that the statement did not apply to the participant and 3 indicates that it applied very much or most of the time. Scores for each subscale were calculated by summing the relevant 14 items, with higher scores indicating greater symptom severity. Severity levels were interpreted according to established DASS-42 cut-off scores, as reported in previous research (Lovibond and Lovibond, 1995; Liasi et al., 2021).

- Depression: 0–9 (Normal), 10–13 (Mild), 14–20 (Moderate), 21–27 (Severe), 28 + (Extremely severe)
- Anxiety: 0–7 (Normal), 8–9 (Mild), 10–14 (Moderate), 15–19 (Severe), 20 + (Extremely severe)
- Stress: 0–14 (Normal), 15–18 (Mild), 19–25 (Moderate), 26–33 (Severe), 34 + (Extremely severe)

### 2.4 Linguistic and cultural review

Because the DASS-42 is an established instrument, the study relied on the original scale and previously published validation evidence for the Arabic version. In the present study, the Arabic wording was reviewed by four experts in psychology and education to ensure clarity, cultural appropriateness, and suitability for Palestinian undergraduate students. Minor wording adjustments were made where needed, without changing the original response format or scoring structure.

### 2.5 Reliability

To determine internal consistency, the DASS-42 was piloted among a small group of undergraduate students who were not part of the study sample. Cronbach’s alpha coefficients indicated acceptable reliability levels for the three sub-scales: Depression (*α* = 0.79), Anxiety (*α* = 0.76), and Stress (*α* = 0.80). The overall reliability coefficient for the entire scale was α = 0.78, providing evidence that the instrument had acceptable internal consistency for research use.

### 2.6 Data analysis

Data were analysed using SPSS version 26. Descriptive statistics, including frequencies and percentages, were used to summarise participants’ demographic characteristics and the severity levels of depression, anxiety, and stress. Depression, anxiety, and stress total scores were calculated by summing the relevant 14 items for each DASS-42 subscale.

Before conducting inferential analyses, normality was assessed using Shapiro–Wilk tests and visual inspection of histograms and Q-Q plots. The assumptions of the non-parametric tests were checked before analysis. Each participant contributed one independent response, the grouping variables were categorical, and the depression, anxiety, and stress scores were treated as continuous DASS-42 subscale scores. Since the score distributions deviated from normality, non-parametric tests were considered appropriate. Mann–Whitney U tests were conducted to compare depression, anxiety, and stress scores according to gender and faculty type. Kruskal–Wallis H tests were conducted to examine differences across GPA categories. Where a statistically significant Kruskal–Wallis H test result was found, post-hoc pairwise comparisons with adjusted significance values were conducted to identify which GPA groups differed significantly.

Effect sizes were reported using r for Mann–Whitney U tests and epsilon-squared (ε^2^) for Kruskal–Wallis H tests. Statistical significance was set at *p* < 0.05.

## 3 Results

### 3.1 Demographic variables among students

Table 1 presents the demographic characteristics of the study participants. The sample consisted of 384 undergraduate students at the Arab American University, including 204 males (53.12%) and 180 females (46.88%). Most participants were enrolled in arts faculties (54.95%), while 45.05% were enrolled in scientific faculties. Regarding GPA, the largest group had a GPA between 3.00 and 3.66 (35.42%), followed by 3.67–4.00 (29.43%), 2.33–2.99 (26.04%), and 2.00–2.32 (9.11%).

**Table 1:** Demographic characteristics of the study participants.

Demographic variable	Category	Frequency	Percentage
GPA	2.00–2.32	35	9.11%
	2.33–2.99	100	26.04%
	3.00–3.66	136	35.42%
	3.67–4.00	113	29.43%
	Total	384	100.00%
Faculty type	Arts faculties	211	54.95%
	Scientific faculties	173	45.05%
	Total	384	100.00%
Gender	Male	204	53.12%
	Female	180	46.88%
	Total	384	100.00%

This study examined the levels of depression among students at the Arab American University. As shown in Table 2, a substantial proportion of students were classified within the severe and extremely severe depression categories, as the percentage of those classified within the extremely severe depression category reached 67.45%, followed by the category of severe depression by 25.26%, while the lowest percentages were among the moderate 5.73% and mild 1.04% categories, while the percentage of normal was only 0.52%. These results indicate a very high prevalence of depression among students.

**Table 2:** Distribution of depression severity levels based on the DASS-42.

Depression severity level	Frequency	Percentage
Normal	2	0.52%
Mild	4	1.04%
Moderate	22	5.73%
Severe	97	25.26%
Extremely severe	259	67.45%
Total	384	100.00%

**Figure 1:**
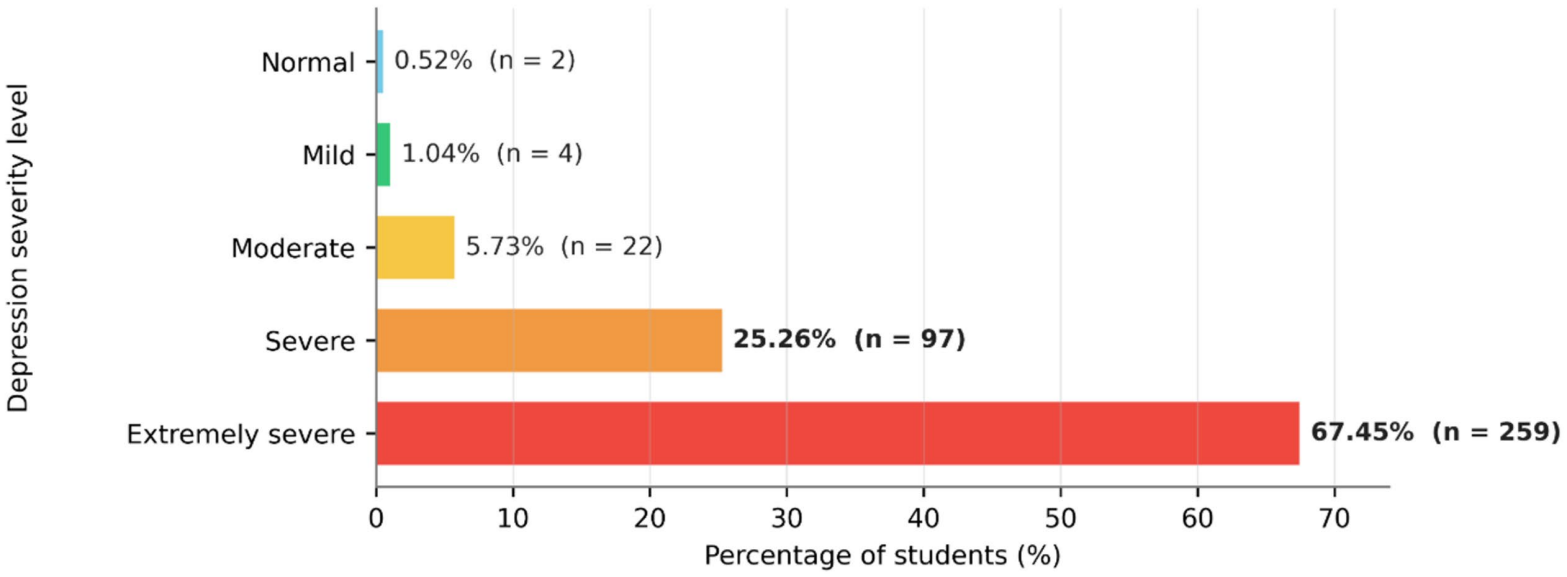
Distribution of depression severity levels among undergraduate students based on the DASS-42. Percentages are based on the total sample (*n* = 384).

Figure 1 illustrates the distribution of depression severity levels among undergraduate students. The findings show that most students were classified within the extremely severe depression category (67.45%, *n* = 259), followed by the severe category (25.26%, *n* = 97). Smaller proportions were classified as moderate (5.73%, *n* = 22), mild (1.04%, *n* = 4), or normal (0.52%, *n* = 2), indicating a notably high burden of depressive symptoms among the study sample.

This study examined the levels of anxiety among students at the Arab American University. As shown in Table 3, severe anxiety is the most prevalent among students at 33.33%, followed by moderate anxiety at 22.40%, extremely severe anxiety at 20.83%, mild anxiety at 13.54%, while the rate of normal was 9.90%. These results indicate elevated anxiety symptoms among the study sample.

**Table 3:** Distribution of anxiety severity levels based on the DASS-42.

Anxiety severity level	Frequency	Percentage
Normal	38	9.90%
Mild	52	13.54%
Moderate	86	22.40%
Severe	128	33.33%
Extremely severe	80	20.83%
Total	384	100.00%

**Figure 2:**
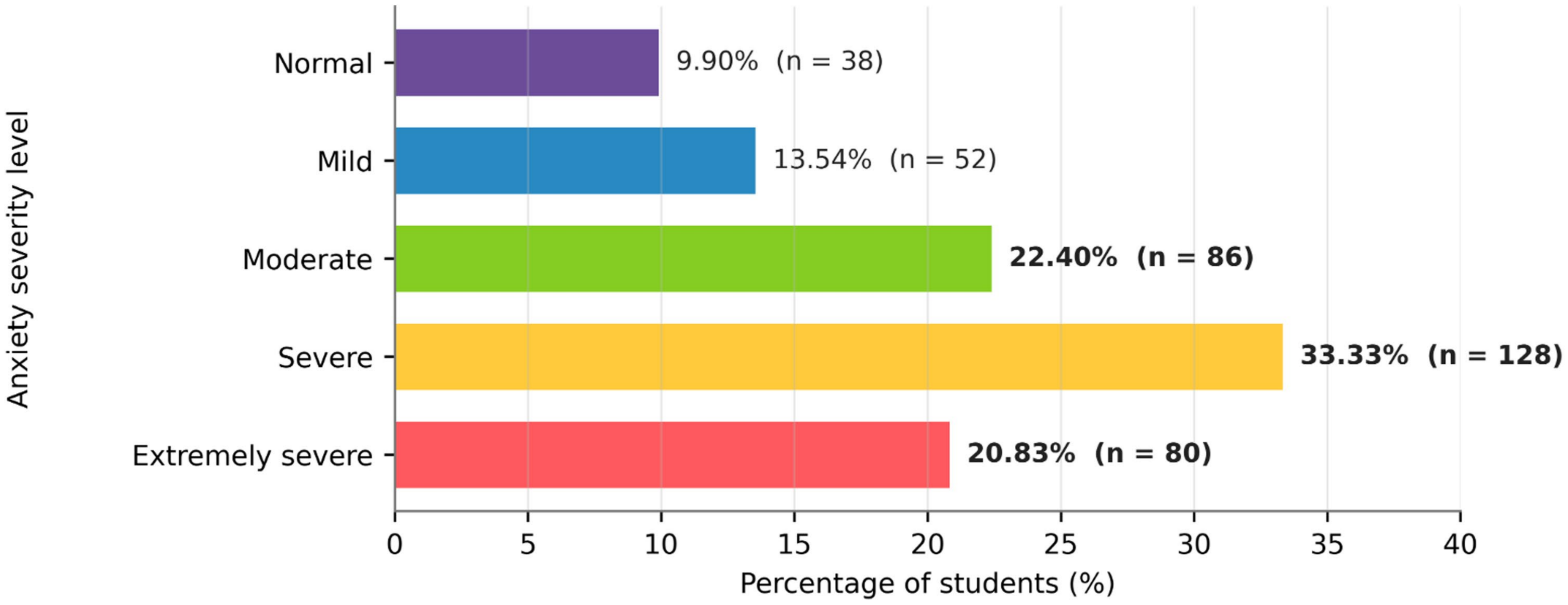
Distribution of anxiety severity levels among undergraduate students based on the DASS-42. Percentages are based on the total sample (*n* = 384).

Figure 2 shows that severe anxiety was the most common category among students (33.33%, *n* = 128), followed by moderate anxiety (22.40%, *n* = 86) and extremely severe anxiety (20.83%, *n* = 80). Mild anxiety was reported by 13.54% of students (*n* = 52), while 9.90% (*n* = 38) were classified within the normal range.

This study examined the levels of stress among students at the Arab American University. As shown in Table 4, the percentage of students classified within the severe stress category reached 44.92%, followed by the category of extremely severe stress by 32.03%, while the moderate percentage was 14.32%, mild 4.69%, and normal only 1.04%. This suggests that the psychological distress among students is high and highlights the need for appropriate university-based mental health support.

**Table 4:** Distribution of stress severity levels based on the DASS-42.

Stress severity level	Frequency	Percentage
Normal	4	1.04%
Mild	18	4.69%
Moderate	55	14.32%
Severe	184	44.92%
Extremely severe	123	32.03%
Total	384	100.00%

**Figure 3:**
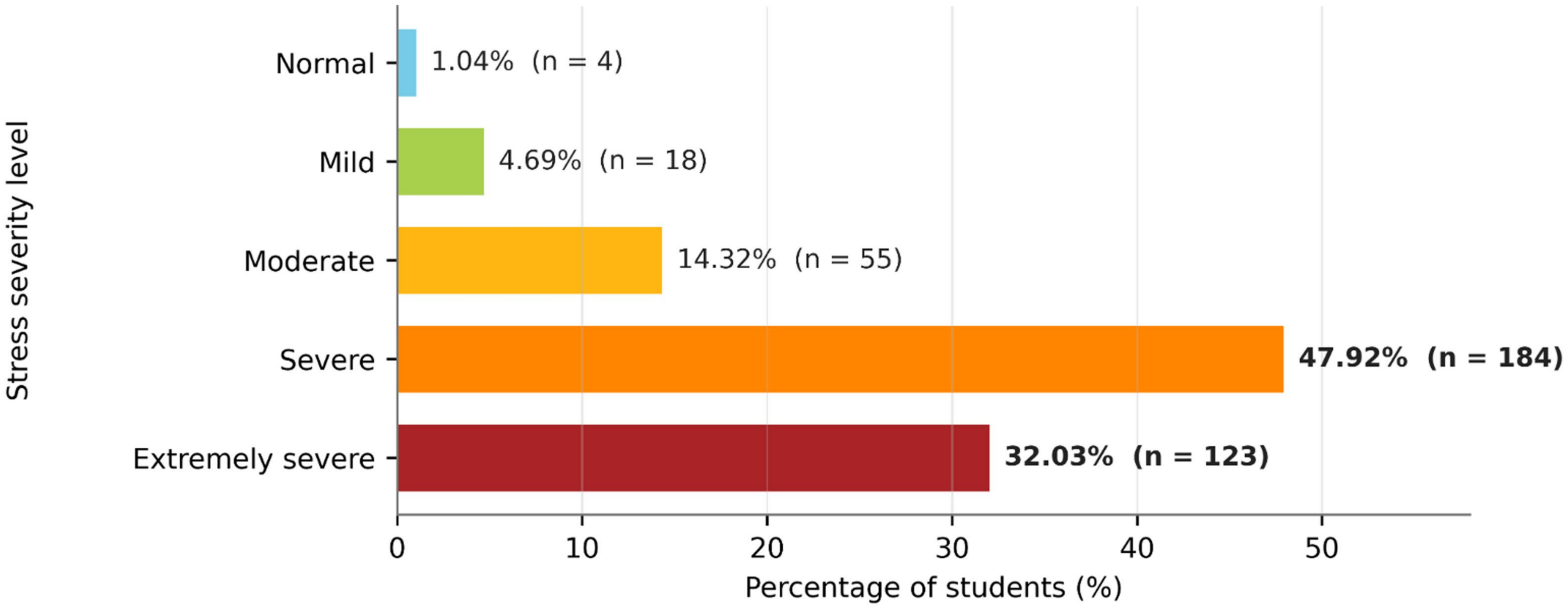
Distribution of stress severity levels among undergraduate students based on the DASS-42. Percentages are based on the total sample (*n* = 384).

Figure 3 shows that severe stress was the most common category among students (44.92%, *n* = 184), followed by extremely severe stress (32.03%, *n* = 123). Moderate stress was reported by 14.32% of students (*n* = 55), while smaller proportions were classified as mild (4.69%, *n* = 18) or normal (1.04%, *n* = 4).

### 3.2 Inferential analysis

Before conducting group comparisons, normality was assessed for depression, anxiety, and stress total scores. Since the scores were not normally distributed, non-parametric tests were used. Mann–Whitney U tests were used for comparisons by gender and faculty type, while Kruskal–Wallis H tests were used for comparisons across GPA categories.

Normality assessment indicated that depression, anxiety, and stress scores were not normally distributed. As shown in Table 5, Shapiro–Wilk tests were statistically significant for depression (W = 0.922, *p* < 0.001), stress (W = 0.966, *p* < 0.001), and anxiety (W = 0.957, *p* < 0.001). Visual inspection of histograms and Q-Q plots also showed deviations from normality. Therefore, non-parametric tests were used for group comparisons.

**Table 5:** Normality assessment for depression, anxiety, and stress scores.

Variable	Mean	SD	Median	IQR	Skewness	Kurtosis	Shapiro–Wilk W	*p*-value	Decision
Depression score	27.15	6.82	29	9	−1.095	1.570	0.922	*p* < 0.001	Non-normal
Stress score	26.67	5.77	28	8	−0.595	−0.002	0.966	*p* < 0.001	Non-normal
Anxiety score	26.82	6.32	28	9	−0.595	−0.260	0.957	*p* < 0.001	Non-normal

SD, standard deviation; IQR, interquartile range.

As shown in Table 6, Mann–Whitney U tests indicated a statistically significant gender difference in depression scores, U = 15,515.00, Z = −2.62, *p* = 0.009, *r* = 0.134, with male students showing higher mean ranks than female students. No statistically significant gender differences were found for anxiety or stress scores. Similarly, no statistically significant differences were found in depression, anxiety, or stress scores according to faculty type. Overall, the effect sizes were negligible to small.

**Table 6:** Mann–Whitney U test results by gender and faculty type.

Grouping variable	Outcome	Group	*N*	Mean rank	Sum of ranks	U	Z	*p*-value	*r*
Gender	Depression	Male	204	206.45	42,115.00	15,515.00	−2.62	0.009	0.134
		Female	180	176.69	31,805.00				
Gender	Anxiety	Male	204	200.74	40,951.50	16,678.50	−1.55	0.121	0.079
		Female	180	183.16	32,968.50				
Gender	Stress	Male	204	201.16	41,037.00	16,593.00	−1.63	0.104	0.083
		Female	180	182.68	32,883.00				
Faculty type	Depression	Scientific faculties	173	195.82	33,877.00	17,677.00	−0.53	0.596	0.027
		Arts faculties	211	189.78	40,043.00				
Faculty type	Anxiety	Scientific faculties	173	200.64	34,711.00	16,843.00	−1.30	0.193	0.066
		Arts faculties	211	185.82	39,209.00				
Faculty type	Stress	Scientific faculties	173	194.90	33,718.00	17,836.00	−0.38	0.701	0.020
		Arts faculties	211	190.53	40,202.00				

*r* = effect size for the Mann–Whitney U test.

**Figure 4:**
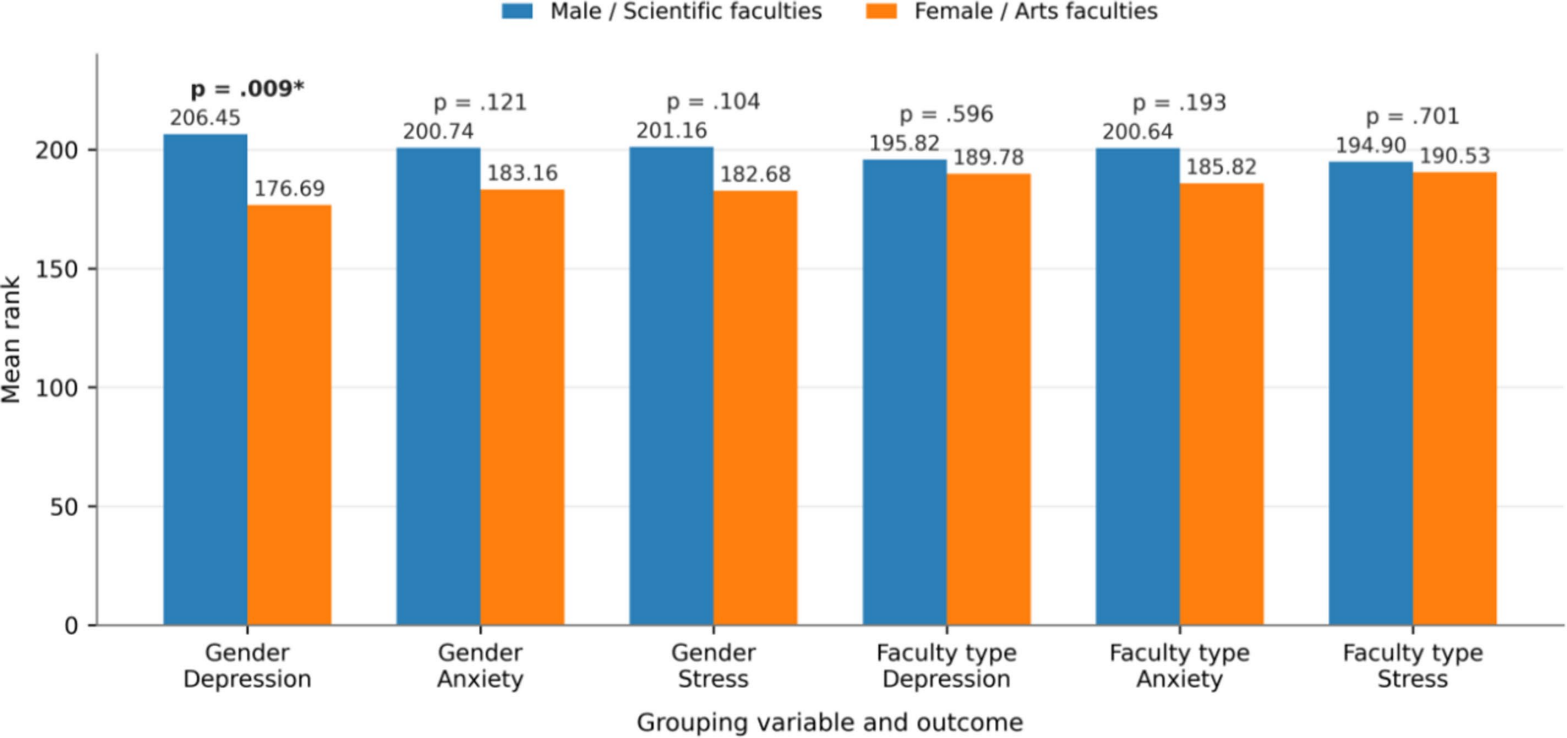
Mean ranks of depression, anxiety, and stress scores by gender and faculty type. Mean ranks are based on Mann-Whitney U tests. *p < 0.05.

Figure 4 presents the mean ranks of depression, anxiety, and stress scores by gender and faculty type. Male students showed higher mean ranks than female students across the three outcomes; however, only the gender difference in depression scores was statistically significant (*p* = 0.009). Differences by faculty type were not statistically significant for depression, anxiety, or stress.

Kruskal–Wallis H tests were conducted to examine differences in depression, anxiety, and stress scores across GPA categories. As shown in Tables 7, a statistically significant difference was found for depression scores across GPA groups, H(3) = 8.21, *p* = 0.042, ε^2^ = 0.014, indicating a small effect. The highest mean rank for depression was observed among students with a GPA of 3.67–4.00, while the lowest mean rank was observed among students with a GPA of 2.33–2.99. No statistically significant differences were found for anxiety, H(3) = 4.96, *p* = 0.175, ε^2^ = 0.005, or stress, H(3) = 6.99, *p* = 0.072, ε^2^ = 0.010.

**Table 7:** Kruskal–Wallis H Test results by GPA category.

Outcome	GPA category	*N*	Mean rank	H	df	p-value	ε^2^
Depression	2.00–2.32	35	184.70	8.21	3	0.042	0.014
	2.33–2.99	100	167.75				
	3.00–3.66	136	198.66				
	3.67–4.00	113	209.41				
Anxiety	2.00–2.32	35	169.64	4.96	3	0.175	0.005
	2.33–2.99	100	178.51				
	3.00–3.66	136	197.82				
	3.67–4.00	113	205.56				
Stress	2.00–2.32	35	156.40	6.99	3	0.072	0.010
	2.33–2.99	100	185.72				
	3.00–3.66	136	192.05				
	3.67–4.00	113	210.23				

ε^2^ = epsilon-squared effect size.

**Figure 5:**
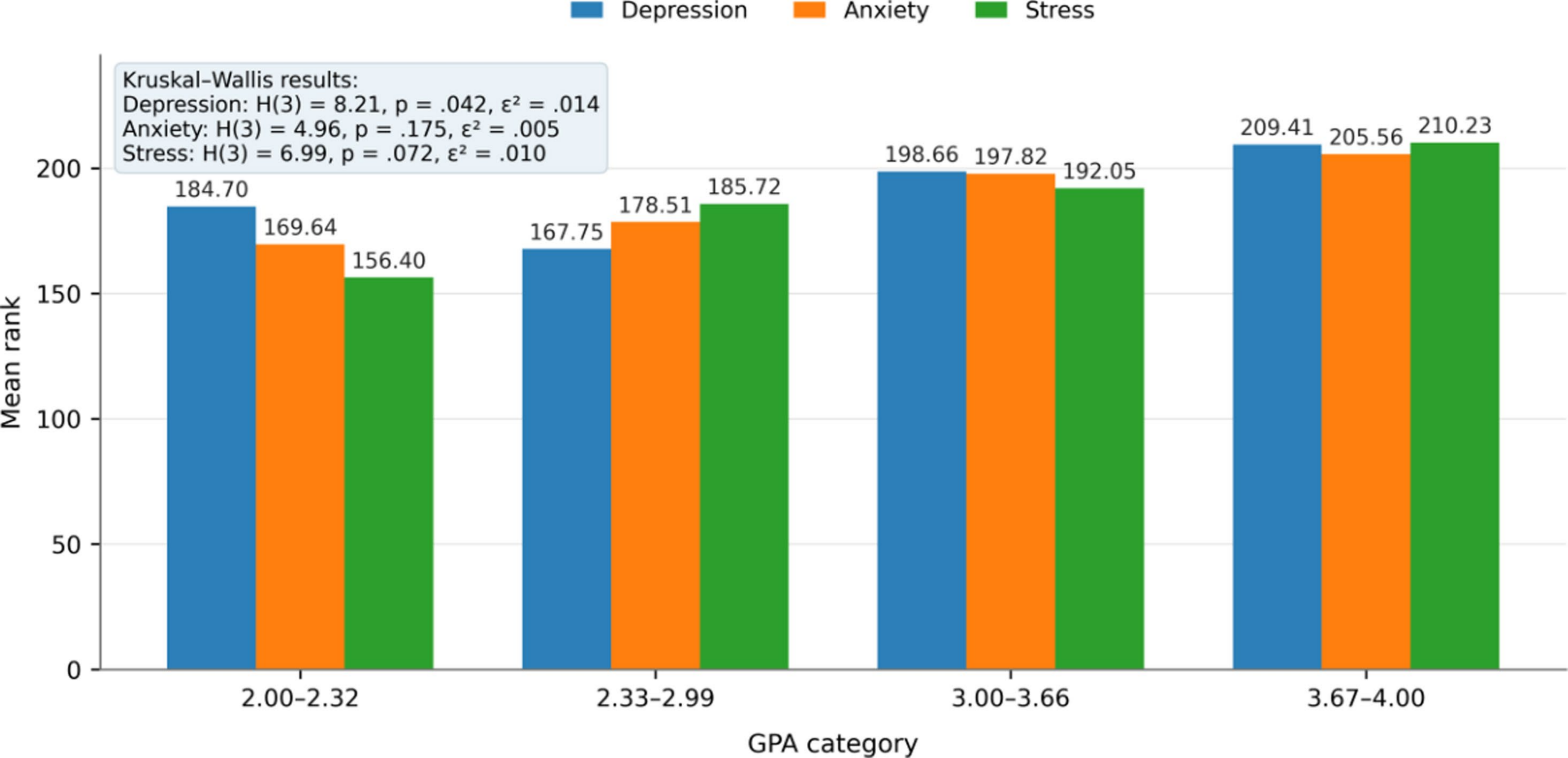
Mean ranks of depression, anxiety, and stress scores by GPA category. Mean ranks are based on Kruskal–Wallis H tests. Only depression differed significantly across GPA categories (*p* < 0.05).

Figure 5 presents the mean ranks of depression, anxiety, and stress scores across GPA categories. Depression scores differed significantly across GPA groups, with the highest mean rank observed among students with a GPA of 3.67–4.00 and the lowest mean rank among students with a GPA of 2.33–2.99. Anxiety and stress scores did not differ significantly across GPA categories.

As shown in Table 8, post-hoc pairwise comparisons were conducted for depression scores across GPA categories because the Kruskal–Wallis H test was statistically significant. After adjustment for multiple comparisons, only the difference between students with a GPA of 2.33–2.99 and those with a GPA of 3.67–4.00 remained statistically significant (adjusted *p* = 0.037). Students with a GPA of 3.67–4.00 had a higher mean rank for depression scores than students with a GPA of 2.33–2.99. No other pairwise comparisons were statistically significant.

**Table 8:** *Post-hoc* pairwise comparisons for depression scores by GPA category.

Pairwise comparison	Test statistic	Std. error	Std. test statistic	*p*-value	Adjusted *p*-value
2.33–2.99 vs. 2.00–2.32	−16.955	21.766	−0.779	0.436	1.000
2.33–2.99 vs. 3.00–3.66	−30.917	14.599	−2.118	0.034	0.205
2.33–2.99 vs. 3.67–4.00	−41.662	15.216	−2.738	0.006	0.037
2.00–2.32 vs. 3.00–3.66	−13.962	21.006	−0.665	0.506	1.000
2.00–2.32 vs. 3.67–4.00	−24.707	21.439	−1.152	0.249	1.000
3.00–3.66 vs. 3.67–4.00	−10.745	14.107	−0.762	0.446	1.000

Adjusted *p*-values are reported for multiple pairwise comparisons. Statistical significance was set at *p* < 0.05.

## 4 Discussion

The findings of the present study indicate a substantial burden of psychological distress among undergraduate students at the Arab American University. According to the DASS-42 severity levels, 67.45% of the students were classified within the extremely severe depression category, while 25.26% were found to be severe. The anxiety levels were also relatively high because 33.33% were classified as having severe anxiety, while 20.83% of students experienced extremely severe anxiety. Moreover, stress levels were also elevated since 44.92% were classified as severe, with 32.03% being extremely severe. The results show that depression, anxiety, and stress are serious mental health concerns in the sample. However, because the DASS-42 is a self-report screening tool, the results should be interpreted as indicators of symptom severity rather than clinical diagnoses.

The findings complement earlier research from Palestine which reported high levels of psychological distress among college students. Ahmed et al. (2024) reported high prevalence rates of depression and anxiety among Palestinian students, while Al-Dabbour et al. (2025), Amro (2024), Ghanim et al. (2022), and Amro et al. (2025) documented elevated levels of psychological distress in students in the Gaza Strip and the West Bank. The findings of this study thus provide further evidence demonstrating that student mental health in Palestine may be understood in relation to the complex interplay between academic demands, socio-political instability, economic difficulties, exposure to violence, disrupted educational conditions and limited access to psychological support.

The results for demographic variables indicated that male students had significantly higher mean ranks for depression scores than female students, while no significant gender differences were found for anxiety or stress. Moreover, faculty type did not show any significant association with depression, anxiety, or stress suggesting that psychological distress was not limited to a specific academic discipline. These findings partially align with Ahmed et al. (2024) regarding the absence of gender differences in anxiety, but differ regarding depression, where the present study found higher depression mean ranks among male students (Al-Dabbour et al., 2025; Bayram and Bilgel, 2008; Dyrbye et al., 2006). The reasons for differences may be different sample characteristics, timing of data collection, cultural context, and severity of academic and political stressors.

Regarding academic performance, the Kruskal–Wallis H test highlighted a statistically significant difference in depression scores across GPA categories, even though its effect size is small. Post-hoc pairwise comparisons revealed that the only significant difference was between the students with a GPA of 2.33–2.99 and those with a GPA of 3.67–4.00, with the higher GPA group showing a higher mean rank for depression scores. There were no significant differences in anxiety and stress based on GPA category. This indicates a complex relationship between depression and academic performance which should not be interpreted causally. High-achieving students may be facing more pressure to succeed academically, but on the other hand, depression can also impact students’ motivation, concentration and performance. This supports previous literature highlighting the close relationship between mental wellbeing and educational outcomes (Pascoe et al., 2020; World Health Organization, 2022).

Although mindfulness was not empirically examined in the present study, it can be viewed as a potentially helpful technique for students facing elevated depression, anxiety, and stress. Previous studies indicate that mindfulness techniques can help students manage their emotions, enhance present-moment awareness, and cope with problems (Galante et al., 2018; de Carvalho et al., 2017; Serrão et al., 2022; Laranjeira and Querido, 2022). In Palestine, mindfulness may be integrated within broader institutional support, such as counselling services, early screening, psychoeducation, academic advising, peer-support programmes, and referral pathways for students with severe symptoms.

Overall, the findings emphasise that Palestinian universities should regard student mental health as an important concern in the planning of higher education. Universities should provide accessible and culturally sensitive counselling services, screen for mental health conditions on a regular basis, offer stress-management and resilience training, and train the academic staff in recognising the signs of psychological distress. Future studies should include students from multiple Palestinian universities, apply longitudinal and mixed-method designs and examine additional factors affecting students’ mental health, along with the effects of trauma, family support, financial difficulties, coping strategies, and access to mental health services.

## 5 Recommendations

1 Palestinian universities should strengthen accessible and culturally responsive psychological counselling services on campus to support students experiencing depression, anxiety, and stress. These services should include early screening, individual counselling, referral pathways, and follow-up support for students with severe symptoms.
2 Academic counselling should also be integrated with psychological support, particularly for students who may experience both academic pressure and depressive symptoms. The significant association between GPA category and depression highlights the need for coordinated academic-psychological support rather than addressing academic performance separately from mental health.
3 Universities should provide preventive workshops and training programmes that focus on emotional regulation, coping strategies, time management, stress management, resilience, and help-seeking behaviours. Mindfulness-based and emotional intelligence-based activities may also be incorporated as supportive approaches within broader mental health programmes.
4 Partnerships between universities, health institutions, and non-governmental organisations should be strengthened to secure resources for student mental health services, especially during periods of socio-political crisis and educational disruption.

## 6 Limitations

This study has several limitations that should be considered when interpreting its findings. First, the cross-sectional design limits the ability to establish causal relationships between socio-political crisis conditions and students’ levels of stress, anxiety, and depression. Therefore, the results should be interpreted as associations rather than evidence of causality. Second, the study relied on self-reported data, which may be affected by recall bias, social desirability bias, or participants’ willingness to disclose psychological symptoms. Third, although the DASS-42 is a widely used screening instrument, this study did not include clinical interviews, medical records, or multi-source verification to confirm mental health diagnoses. Fourth, the sample included undergraduate students from the Arab American University only, which may restrict the generalisability of the findings to students in other Palestinian universities or different cultural and institutional contexts. Finally, the study did not examine other psychological, social, or environmental factors, such as family support, coping strategies, trauma exposure, financial hardship, or academic resilience, which may have influenced students’ psychological wellbeing. Future research should use longitudinal and mixed-method designs to examine changes in students’ mental health over time and to provide a deeper understanding of the factors contributing to psychological distress in crisis-affected higher education contexts.

## 7 Conclusion

According to the findings of the study, the undergraduate students at the Arab American University experience elevated levels of stress, anxiety, and depression, indicating that their mental health challenges extend beyond ordinary academic stress. Although the study did not examine the causes of these conditions, these findings may be understood in relation to the ongoing socio-political instability, financial hardship, and increasing academic pressure paired with limited psychological support within university settings.

This evidence highlights the need for Palestinian universities to develop a comprehensive institutional strategy aimed at enhancing students’ mental health and well-being. Such a strategy should include the establishment of psychological counselling services and mental health awareness programmes that are grounded in transformative learning, emotional intelligence, and mindfulness principles.

Greater attention to university students’ mental health in Palestine represents an investment in human capital, educational quality, and psychological resilience during ongoing periods of crisis.

## Data Availability

The raw data supporting the conclusions of this article will be made available by the authors, without undue reservation.
